# Cross-cultural comparison of motor competence in children from Australia and Belgium

**DOI:** 10.3389/fpsyg.2015.00964

**Published:** 2015-07-13

**Authors:** Farid Bardid, James R. Rudd, Matthieu Lenoir, Remco Polman, Lisa M. Barnett

**Affiliations:** ^1^Department of Movement and Sports Sciences, Ghent UniversityGhent, Belgium; ^2^Institute of Sport, Exercise and Active Living, Victoria UniversityMelbourne, VIC, Australia; ^3^Psychology Department, Bournemouth UniversityDorset, UK; ^4^Faculty of Health, Deakin UniversityMelbourne, VIC, Australia

**Keywords:** motor competence, motor assessment, KTK, children, cross-cultural comparison, Belgium, Australia

## Abstract

Motor competence in childhood is an important determinant of physical activity and physical fitness in later life. However, childhood competence levels in many countries are lower than desired. Due to the many different motor skill instruments in use, children's motor competence across countries is rarely compared. The purpose of this study was to evaluate the motor competence of children from Australia and Belgium using the Körperkoordinationstest für Kinder (KTK). The sample consisted of 244 (43.4% boys) Belgian children and 252 (50.0% boys) Australian children, aged 6–8 years. A MANCOVA for the motor scores showed a significant country effect. Belgian children scored higher on jumping sideways, moving sideways and hopping for height but not for balancing backwards. Moreover, a Chi squared test revealed significant differences between the Belgian and Australian score distribution with 21.3% Belgian and 39.3% Australian children scoring “below average.” The very low levels reported by Australian children may be the result of cultural differences in physical activity contexts such as physical education and active transport. When compared to normed scores, both samples scored significantly worse than children 40 years ago. The decline in children's motor competence is a global issue, largely influenced by increasing sedentary behavior and a decline in physical activity.

## Introduction

The ability to perform various motor skills (e.g., running, kicking, jumping, throwing) in a proficient manner, is often defined as motor competence (Gabbard, 2008; Haga et al., 2008; Gallahue et al., 2012). Motor competence relies on motor coordination which refers to the cooperation between muscles or muscle groups to produce a purposeful action or movement (Magill, 2011), and physical fitness which refers to the capacity to perform physical activity (Ortega et al., 2008).

Over the past few decades, decreased levels of motor competence in primary school children have been reported in Western countries (Bös, 2003; Okely and Booth, 2004; Vandorpe et al., 2011; Hardy et al., 2013; Tester et al., 2014). These findings are of major concern as children with high motor competence have been linked with positive outcomes in both physical activity (PA) and weight status (Lubans et al., 2010). Furthermore, motor competence predicts levels of PA and physical fitness in later life (Barnett et al., 2008; Lopes et al., 2011; Jaakkola et al., 2015). In view of this, it is important to examine and monitor motor behavior during childhood in order to provide appropriate strategies to support children's motor development.

A variety of test instruments are used to measure motor competence during childhood (see Cools et al., 2009 and Wiart and Darrah, 2001 for reviews on this matter). The choice of assessment batteries depends on a number of criteria such as the purpose of measurement, age specificity, and the suitability of the test for the target group (Cools et al., 2009). The popularity and implementation of test instruments also varies depending on the geographical region. For example, in Australia, assessment batteries such as the Test of Gross Motor Development, Second Edition (TGMD-2; Ulrich, 2000), are generally used to measure motor competence of children through a set of fundamental motor skills (e.g., running, throwing, jumping, catching), whilst Belgium and other European countries have preferred to use Körperkoordinationstest für Kinder (KTK; Kiphard and Schilling, 1974, 2007), a non-sport specific assessment of a child's gross motor coordination.

Although motor tests measure the same broad construct (i.e., motor competence), research on test comparisons generally reveals only moderate correlations. For instance, a study of Fransen et al. (2014) compared the KTK and Bruininks-Oseretsky Test of Motor Proficiency, Second Edition (BOT-2; Bruininks and Bruininks, 2005) in primary school children and found a moderate association between the two tests performances. These findings are similar to other convergent validity studies (Smits-engelsman et al., 1998; Van Waelvelde et al., 2007; Logan et al., 2011) which suggests that assessment batteries should not be used interchangeably to evaluate motor competence. Alternatively, the wide adoption of a highly standardized test battery, would enable comparison of motor competence within and between countries.

There is a dearth of research comparing children's motor competence between countries. One study by Chow et al. (2001) compared the motor competence between children from China (Hong Kong) and the United States, and revealed differences between the groups: Chinese children performed significantly better on manual dexterity and balance tasks whilst American children outperformed Chinese children on throwing and catching tasks. These differences give insight into different cultural practices (such as encouragement in some types of sport e.g., baseball in America) that help or hinder development in certain types of skills. Clearly, cross-cultural research can provide valuable insights into how different motor skills are developed in different cultural contexts and how tests which measure specific motor skills are sensitive to cultural differences.

In summary, it would be unwise to undertake comparisons using different assessment tools because the small, but significant, differences in measurement might not provide meaningful findings and valid conclusions. As highlighted in the study of Chow et al. (2001), we should also be cautious about using an assessment tool which relates more closely to the sports played in some countries more than others, as whilst this gives information on particular skills it may not present an overall picture of the populations' motor competence. A better approach would be to adopt a standardized non sport specific test of motor competence across all countries. The KTK assesses motor coordination without a sport context and may therefore be such a suitable test. It is a standardized and popular test battery that makes it an appropriate tool to measure motor competence internationally and provide cross-cultural comparisons (Iivonen et al., 2014).

There is evidence of streamlining of assessment and international collaborations in other areas of health and physical activity behavior. An example is the development of the International Physical Activity Questionnaire (IPAQ) (Craig et al., 2003). In 1998 an International Consensus Group met in Geneva with the purpose of developing a self-reported measure of physical activity which could be used to assess physical activity across countries. It was recognized at that time that physical inactivity was a global health concern, but that there were no standardized approaches to measurement which made international comparisons and global surveillance challenging. Similarly, the wide adoption of a single test to measure motor competence, has the potential to build a strong picture of how children are performing on an international level rather than just on a national level. This will have many benefits in terms of understanding on a global level how motor competent children are and then proceeding to understand what cultural factors help to better facilitate motor competence.

The aim of this study was to evaluate the motor competence of 6 to 8 year-old children from Australia and Belgium using the Körperkoordinationstest für Kinder (KTK). A secondary aim of this study was to compare the distribution of both samples across the KTK performance categories and against the reference population from 1974. Based on the declining levels of motor competence found in Western countries (Bös, 2003; Okely and Booth, 2004; Vandorpe et al., 2011; Hardy et al., 2013; Tester et al., 2014), it was hypothesized that the distribution of both Australian and Belgian children would be shifted toward the lower end of the motor competence continuum when compared to the KTK reference population of 1974.

## Method

### Participants

Data were collected in Melbourne (Australia) between October 2012 and June 2013 and Flanders (Belgium) between September 2012 and November 2012. A total of 496 children (252 Australian and 244 Belgian children) between the ages of 6 and 8 years participated. In Melbourne, four schools were selected in four local council municipalities. In Flanders, children were recruited from five schools in different provinces. For each participant written informed consent was obtained from the parents or guardian. The study was approved by the University Ethics Committee and the Department of Education and Early Childhood Development in both countries.

### Measurements

All assessments were conducted by trained assessors. All assessors had a Physical Education background and followed a training on KTK assessment. For the tests, children were barefooted and wore light sport clothes. First, anthropometric measurements (height and weight) were taken. Secondly, children's motor competence was assessed with the KTK.

### Anthropometry

In both countries, height and weight were measured with an accuracy of 0.1 cm and 0.1 kg, respectively. In Australia, height was assessed with a Mentone PE087 portable stadiometer (Mentone Educational Centre, Melbourne, Australia) and weight was assessed using a SECA 761 balance scale (SECA GmbH & Co. KG., Birmingham, UK). In Belgium, height was measured by means of a SECA 123 portable stadiometer (SECA GmbH & Co. KG., Hamburg, Germany) and weight was measured using a SECA Robusta 813 digital balance scale (SECA GmbH & Co. KG, Hamburg, Germany). Height and weight values were used to calculate body mass index (BMI) [BMI = weight (kg)/height^2^ (m^2^)]. Weight status was determined by the sex- and age-specific BMI cut-off values for children of the International Obesity Task Force (Cole and Lobstein, 2012).

### Gross motor coordination

The KTK measures gross motor coordination in typically and atypically developing children, aged 5–14 years (Kiphard and Schilling, 1974, 2007). The psychometric quality of the KTK is good. Content and construct validity have been established for the general pediatric population (Kiphard and Schilling, 1974, 2007). The test manual also describes good-to-excellent test-retest and inter-rater reliability (all *r* > 0.85) as well as good intraclass correlations for all test items (*r* = 0.80–0.96).

In both countries the KTK was administered according to the manual guidelines (Kiphard and Schilling, 1974, 2007). The KTK consists of 4 outcome-based subtests. Walking backwards (WB) requires participants to walk backwards along three different balance beams, with increasing levels of difficulty due to the width of the beams decreasing from 6 to 4.5 to 3 cm, respectively. Three trials are given for each balance beam with a maximum score of 72 steps (i.e., maximum 8 steps per trial). Hopping for height (HH) requires participants to hop on one leg over an increasing number of 5 cm foam blocks to a maximum of 12 blocks. Participants have to begin hopping 1.5 m away from the foam blocks, hop up to and over the foam block and complete a further two hops for the trial to be deemed successful. Three trials are given for each height with 3, 2, or 1 point(s) given for a successful performance during 1st, 2nd, or 3rd trial, respectively. Jumping sideways (JS) requires participants to complete as many sideways jumps as they can, with feet together, over a wooden slat in 15 s. Moving sideways (MS) requires participants to move across the floor during 20 s using two wooden platforms. Participants step from one platform to the next, move the first platform, step on to it, and repeat the same process as much as possible in 20 s. Two trials are given for both jumping sideways and moving sideways. The KTK requires little time to set-up and takes approximately 15–20 min to administer.

Using the normative data of the German 1974 sample, raw item scores were converted into standardized scores adjusting for age (all items) and sex (hopping for height and jumping sideways over a slat). In turn, standardized score items were summed and transformed into a total MQ. The total MQ allows classification of a child's performance into five categories: “impaired” 2%, “poor” 14%, “normal” 68%, “good” 14%, and “high” 2% (Kiphard and Schilling, 1974, 2007).

### Data analysis

Data were analyzed using SPSS Statistics 20 for Windows. Values of *p* ≤ 0.05 were considered statistically significant for all analyses. Descriptive statistics were calculated for anthropometric measures (height, weight, and BMI) and KTK scores (raw and standardized scores). We first investigated whether differences in distribution across BMI categories (based on the International Obesity Task Force cut-off values) were similar for both the Australian and Belgian sample. Further, the effect of country (Australia and Belgium) and age (6–8 years) on KTK raw scores were examined using a 2 × 3 MANCOVA. Since weight status is associated with motor competence (Lubans et al., 2010; D'Hondt et al., 2011), the body mass index (BMI) was included as a covariate in the analysis. Significant interaction and main effects were further investigated with Bonferroni *post-hoc* tests or pairwise comparisons. In addition, the effect of country on the age and sex specific MQs were inspected using One-Way ANCOVAs with BMI as a covariate. Separate models were used for the item MQs and total MQ, i.e., MANCOVA and ANCOVA, respectively. Finally, a chi squared test was used to compare the distributions of Australian and Belgian children across the KTK performance categories (impaired, poor, normal, good, high). Additionally, chi squared analysis was used to compare the observed distribution of both samples with the expected distribution based on the German reference sample.

## Results

### Sample characteristics

Table 1 shows the descriptive statistics of height, weight, and BMI for both Australian and Belgian sample. Chi squared analysis demonstrated that these distributions across BMI categories are similar between both samples (χ^2^ = 6.011; *p* = 0.111; ϕ_c_ = 0.110).

**Table 1:** **Descriptive statistics [Means and standard deviations (M ± SD)] of anthropometric measurements, stratified by age and sex**.

**Age group**	**Variables**	**Australia**		**Belgium**	
		**Boys**	**Girls**	**Boys**	**Girls**
6 years	N	22	23	47	54
	Height (cm)	122.9 ± 5.1	121.9 ± 7.6	120.2 ± 5.8	119.5 ± 6.7
	Weight (kg)	25.2 ± 5.6	24.7 ± 4.5	23.1 ± 4.0	22.9 ± 4.5
	BMI (kg/m^2^)	16.57 ± 2.93	16.55 ± 1.82	15.90 ± 2.00	15.87 ± 1.74
7 years	N	54	55	33	40
	Height (cm)	127.2 ± 5.6	125.9 ± 7.3	129.0 ± 5.8	124.2 ± 5.0
	Weight (kg)	27.4 ± 6.0	26.7 ± 6.5	27.0 ± 5.2	25.3 ± 4.3
	BMI (kg/m^2^)	16.83 ± 2.80	16.64 ± 2.60	16.09 ± 1.89	16.34 ± 2.16
8 years	N	50	47	26	44
	Height (cm)	131.1 ± 6.0	131.6 ± 7.2	133.7 ± 5.7	130.5 ± 6.6
	Weight (kg)	29.5 ± 5.1	30.1 ± 9.0	28.7 ± 3.8	29.0 ± 6.7
	BMI (kg/m^2^)	17.04 ± 2.14	17.15 ± 3.63	16.00 ± 1.43	16.81 ± 2.54
Total	N	126	125	106	138
	Height (cm)	128.0 ± 6.4	127.3 ± 8.1	126.2 ± 8.1	124.4 ± 7.7
	Weight (kg)	27.8 ± 5.7	27.6 ± 7.5	25.7 ± 5.0	25.5 ± 5.8
	BMI (kg/m^2^)	16.87 ± 2.57	16.82 ± 2.91	15.98 ± 1.83	16.31 ± 2.16

### Differences in raw scores between Australian and Belgian children

Mean scores and standard deviations for each country are reported in Table 2. The results of the MANCOVA are presented in Table 3. BMI was shown to be a significant covariate.

**Table 2:** **Means (M) and standard deviations (SD) of performance on the KTK (raw and standardized scores)**.

**Variable**	**Australia *(N = 252)***		**Belgium *(N = 244)***	
	***M***	***SD***	***M***	***SD***
**RAW SCORES**				
Walking backwards	31.1	14.1	27.6	13.1
Hopping for height	34.6	15.0	35.7	15.5
Jumping sideways	44.5	13.8	45.0	12.0
Moving sideways	31.1	7.6	34.5	6.2
**MOTOR QUOTIENTS**				
Walking backwards	88.7	15.3	85.8	13.9
Hopping for height	96.5	17.1	99.5	16.6
Jumping sideways	100.5	17.5	106.6	15.2
Moving sideways	86.0	16.7	97.5	13.9
Total	90.6	16.5	96.4	13.6

**Table 3:** **Interaction and main effects on KTK performance according to country and age group**.

**Variables**	**F_COUNTRY × AGE_**	**η^2^_p_**	**F_COUNTRY_**	**η^2^_p_**	**F_AGE_**	**η^2^_p_**	**F_BMI_*covariate***	**η^2^_p_**
**RAW SCORES**								
Walking backwards	1.42	0.006	2.64	0.005	32.45^***^	0.117	12.39^***^	0.025
Hopping for height	2.97	0.012	8.28^**^	0.017	79.70^***^	0.246	14.88^**^	0.030
Jumping sideways	0.76	0.003	6.61^*^	0.013	71.08^***^	0.226	5.10^*^	0.010
Moving sideways	0.44	0.002	40.52^***^	0.077	26.55^***^	0.098	5.31^*^	0.011

*** p < 0.001,

** p < 0.01,

* *p ≤ 0.05*.

The MANCOVA for the 4 subtests showed a significant country × age effect (Wilks' λ = 0.96; *F* = 2.78; *p* = 0.005; partial η^2^ = 0.022). However, follow-up ANCOVAs could not confirm the interaction effect for any subtest (see Table 3). Results also showed significant main effects for country (Wilks' λ = 0.89; *F* = 14.613; *p* < 0.001; partial η^2^ = 0.108) and age (Wilks' λ = 0.71; *F* = 22.84; *p* < 0.001; partial η^2^ = 0.159). For country effect, significant differences were found for hopping for height, jumping sideways and moving sideways in favor of Belgian children (*p* ≤ 0.01). No significant country differences were found in walking backwards on a balance beam (*p* = 0.105). For age effect, significant differences were found for each subtest with older children performing higher than their 1-year younger counterparts (all *p* ≤ 0.005).

### Comparing Motor Quotients of Australian and Belgian children

Results showed that BMI is a significant covariate in the analyses for the total MQ and all item MQs (*F* ≥ 6.11; *p* ≤ 0.05; η^2^_*p*_ ≤ 0.024) except for jumping sideways (*F* = 2.76; *p* = 0.097; partial η^2^ = 0.026). The ANCOVA for the total KTK Motor Quotient showed a significant country effect (*F* = 13.87; *p* < 0.001; partial η^2^ = 0.027). The performance of Belgian children was higher in comparison with Australian children (see Table 2). The MANCOVA for the Motor Quotients of the subtests showed a significant country effect (Wilks' λ = 0.83; *F* = 25.46; *p* < 0.001; partial η^2^ = 0.172). Motor Quotient scores of Belgian children were significantly higher for jumping sideways (*F* = 14.69; *p* < 0.001; partial η^2^ = 0.029) and moving sideways (*F* = 63.043; *p* < 0.001; partial η^2^ = 0.114) in comparison with Australian children. However, the latter group did score significantly higher on walking backwards (*F* = 6.98; *p* = 0.009; partial η^2^ = 0.014). No significant differences in Motor Quotients were found for hopping for height (*F* = 2.295; *p* = 0.130; partial η^2^ = 0.005).

### KTK classification of motor competence in the Australian and Belgian sample

**Figure 1:**
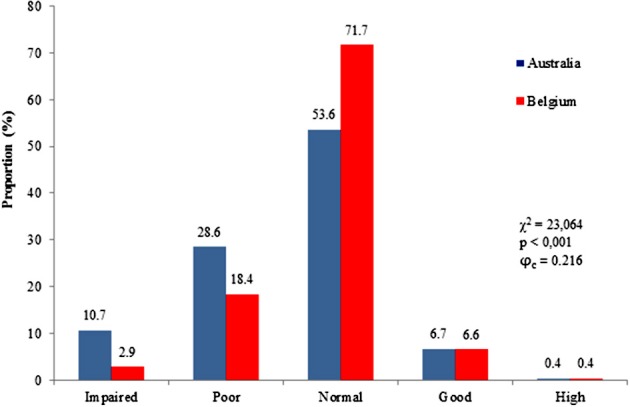
**Proportion of children across KTK performance ratings for both countries**.

The distribution of Australian and Belgian children across the KTK performance categories are shown in Figure 1. A chi-squared analysis demonstrated a significant difference in distribution between both samples (χ^2^ = 23.06; *p* < 0.001; ϕ_c_ = 0.216). The proportion of children scoring in the normal range of motor competence differed between Australia and Belgium (53.6 vs. 71.7%, respectively). Moreover, the percentage of Australian children performing below average was higher compared with Belgian children. The proportion of children scoring above average was similar for the Australian and Belgian sample. Additional chi squared tests also revealed that the observed percentages of both Australian and Belgian across the performance levels differed significantly from the expected percentages of KTK classification based on the German reference sample (Australia: χ^2^ = 90.24; *p* < 0.001; ϕ_c_ = 0.247; Belgium: χ^2^ = 15.68; *p* = 0.003; ϕ_c_ = 0.103). The percentages of Australian and Belgian children scoring below average are 39 and 21%, respectively as opposed to 16% in the German standardization sample. In contrast, the percentages of Australian and Belgian children performing above average are lower compared to the children of the German sample (7.1 vs. 16% and 7 vs. 16%, respectively).

## Discussion

The main aim of this study was to compare the motor competence of 6 to 8 year old children from Australia and Belgium using the KTK. A secondary aim was to compare the Australian and Belgian samples across the different performance categories of the KTK. In view of downward trends of motor competence (Bös, 2003; Okely and Booth, 2004; Vandorpe et al., 2011; Hardy et al., 2013; Tester et al., 2014) we also investigated whether the Australian and Belgian distributions across the KTK categories had shifted toward the lower end of the motor competence spectrum when compared to the KTK reference sample.

Overall, children from Belgium demonstrated a higher level of motor competence. Looking at the raw scores, Belgian children scored significantly better than the Australian children on three of the four individual tasks: moving sideways, jumping sideways and hopping for height. These tasks required a combination of lateral, upper, and lower body coordination. Because this analysis was done using raw scores, the differences between countries at first appeared trivial (see Table 3) however, when the scores were standardized by age and sex, and we looked at the differences between countries using the Motor Quotients, the differences became more meaningful with Belgian children performing 17% higher than Australian children. Looking at the item motor quotients, children from Belgium scored significantly better on two of the four tests, though only one of these can be considered truly meaningful: Belgian children, on average, scored 11% better on moving sideways than Australian children. Australian children performed significantly higher on the walking backwards though the effect size can be regarded as trivial (η^2^_p_ = 0.014) whilst no significant difference was found for hopping for height.

It has been suggested that measuring motor competence (especially when using a product based assessment) also evaluates some elements of a child's physical fitness such as strength, speed, endurance, and flexibility. Our findings might therefore indicate that Belgian children are more fit than Australian children. This may explain why Australian children scored higher on the walking backwards task as this is less sensitive to physical fitness. The other three tests involve both coordination and aspects of physical fitness meaning that physical fitness may be a confounding factor to motor coordination. Results also showed that differences in motor performance between both countries were independent of age. As expected, age was found to influence motor competence within the groups, attesting to the quality of the KTK as a test battery. We also found BMI had a significant negative association in each model reinforcing previous literature on the inverse relationship between weight status and motor competence (D'Hondt et al., 2009; Lubans et al., 2010; Lopes et al., 2012). This points to the importance of adequate motor competence for children's healthy weight status as indicated in the model of Stodden et al. (2008).

In an effort to explain why Australian children generally scored lower than their Belgian counterparts, and why both countries scored significantly lower when compared to German norms, we adopted the three constraints based model as a framework which shapes motor development (Newell, 1986). Descriptive data showed that both samples had similar sex distributions and anthropometric characteristics, although the Belgian children were on average 3 months younger (which is why the difference in raw scores do not appear meaningful as they have not accounted for age). The KTK is a test of gross motor coordination, as such the tasks were novel for all children taking part. It is therefore likely that the PA contexts such as physical education (PE) in pre-school and primary school played a role in the differences observed in the KTK performance.

Early childhood is described as the optimal time to develop motor skills and establish motor competence (Hardy et al., 2010b) and preschool has been lauded as the ideal institution for PA promotion in young children (Ward, 2010; Hinkley et al., 2012). In Belgium, 98% of children aged 3–6 attend a free pre-school program for 30 h a week (Flemish Ministry of Education and Formation, 2011). In Australia, 70% of children aged 3–5 years attend a pre-school program of which only 23% attend for ≥15 h per week, and often there is a cost attached to these services (Pink, 2008). Overall, Australia is performing poorly in its ability to meet a set of minimum standards for children in their formative years when compared to other countries from the Organization for Economic Co-operation and Development. Australia currently only meets two of the 10 standards whilst Belgium complies with six standards (UNICEF Innocenti Research Centre, 2008). Therefore, the lower levels of motor performance observed in Australian children at the age of 6 years may be due to pre-school experiences, or the lack of them prior to beginning primary school.

In both countries, PE may be the main vehicle for developing children's motor competence in primary schools. Differences in policies and common practices in PE may explain the higher motor scores found in Belgian children. The PE curriculum in Flanders is protected by the decree “Education II” (Flemish Ministry, 1990) which legitimizes PE as part of the “basic school curriculum” and dictates that two 50 min lessons a week are compulsory for all children from 6 to 18 years (Arnouts and Spilthoorn, 1999). Though there is little evidence available for the quality of PE, approximately 81% of Flemish primary schools deploy a specialist teacher to teach PE (European Commission/EACEA/Eurydice, 2013). The Australian government recognizes that PE and sporting programs in schools have the potential to make people active for the rest of their lives and one of its primary objectives is to boost the number of children participating in sport through education (Commonwealth of Australia, 2010). However, despite this, PE has been marginalized to the periphery of the school curriculum leading to diminished time on school timetables (Moneghetti, 1993; Morgan and Hansen, 2008; Hardy et al., 2010a). PE in Australian primary schools is generally provided by classroom teachers (Hardy et al., 2010a). However, the total curriculum of the pre-service teacher education—provided by Australian universities—includes only two PE courses (Morgan and Bourke, 2005) which raises questions about the quality of PE in Australian primary schools.

Interestingly, whilst Belgian children displayed better scores overall than Australian children, both groups scored significantly lower than the German standardization sample from 1974. Although this finding could be attributed to cultural differences between these countries, a more likely reason can be found in the international decline in PA over the past decades (Dollman et al., 2005), Australia has seen a 42% decline in active transport between 1971 and 2013 and children's top 10 preferred play spaces have seen a marked transition from outdoors to indoors between 1950 and 2000 (Active Healthy Kids Australia, 2014).

The latter explanation is in line with a large-scale Australian study in primary school children where a general decline was found in motor competence and physical fitness. This decline was especially apparent in 6-year-old children who performed worse than their counterparts in the 1980s in tasks such as underarm throws, catching and bouncing balls (Tester et al., 2014). Lifestyles across Europe and Australia have changed over the past 40 years with advances in technology and increased standards of living and this has have changed how children spend their leisure time with an increase in sedentary activity and a decrease in PA levels (Dollman et al., 2005). In view of Stodden et al. (2008)'s model on the dynamic relationship between the motor competence and PA, the downward trends of PA levels may affect the levels of motor competence and should therefore be addressed by policymakers.

A limitation to this study is the sole focus on gross motor coordination as the measurement of motor competence. However, fundamental motor skills (specifically object control skills) also play a role in children's motor competence and their engagement in physical activity and sports (Barnett et al., 2009), and fitness (Vlahov et al., 2014). Therefore, future research should investigate cross-cultural differences in these fundamental motor skills in order to gain a better understanding of children's motor competence on a global level. Nonetheless, a strength of this study is the use of a standardized and robust assessment tool that is easy to use in both clinical and educational settings (Cools et al., 2009). Importantly, this study has enabled the cross-cultural comparison of motor competence in a large sample of young children.

## Conclusion

This study provides valuable information on cross-cultural comparison of motor competence levels in children using the KTK. Present findings show that overall Belgian children scored generally higher on motor competence than Australian children. Also, distributions across performance categories revealed that a greater percentage of Australian children (nearly twice the Belgian percentage) scored below average. These results can be explained by possible physical activity contexts such as PE and organized sports, however future research is needed to investigate the role of physical activity and fitness on cross-cultural differences in motor competence.

### Conflict of interest statement

The authors declare that the research was conducted in the absence of any commercial or financial relationships that could be construed as a potential conflict of interest.
